# Affective, semantic, frequency, and descriptive norms for 107 face emojis

**DOI:** 10.3758/s13428-024-02444-x

**Published:** 2024-08-15

**Authors:** Tatjana Scheffler, Ivan Nenchev

**Affiliations:** 1 Department for German Language and Literature, Ruhr University Bochum, Universitätsstraße 150, 44801 Bochum, Germany; 2 Department of Psychiatry and Psychotherapy, Charité Campus Mitte, Charité Universitätsmedizin Berlin, Corporate Member of Freie Universität Berlin, Humboldt-Universität zu Berlin, and Berlin Institute of Health at Charité – Universitätsmedizin Berlin, BIH Biomedical Innovation Academy, BIH Charité Digital Clinician Scientist Program, Berlin, Germany

**Keywords:** Emoji, Norming study, Frequency, Arousal, Valence, Word embeddings, Visual complexity, Subjective rating

## Abstract

We introduce a novel dataset of affective, semantic, and descriptive norms for all facial emojis at the point of data collection. We gathered and examined subjective ratings of emojis from 138 German speakers along five essential dimensions: valence, arousal, familiarity, clarity, and visual complexity. Additionally, we provide absolute frequency counts of emoji use, drawn from an extensive Twitter corpus, as well as a much smaller WhatsApp database. Our results replicate the well-established quadratic relationship between arousal and valence of lexical items, also known for words. We also report associations among the variables: for example, the subjective familiarity of an emoji is strongly correlated with its usage frequency, and positively associated with its emotional valence and clarity of meaning. We establish the meanings associated with face emojis, by asking participants for up to three descriptions for each emoji. Using this linguistic data, we computed vector embeddings for each emoji, enabling an exploration of their distribution within the semantic space. Our description-based emoji vector embeddings not only capture typical meaning components of emojis, such as their valence, but also surpass simple definitions and direct emoji2vec models in reflecting the semantic relationship between emojis and words. Our dataset stands out due to its robust reliability and validity. This new semantic norm for face emojis impacts the future design of highly controlled experiments focused on the cognitive processing of emojis, their lexical representation, and their linguistic properties.

## Introduction

Emojis are visual linguistic elements currently widely used in computer-mediated communication. In recent years, a variety of studies have examined their cognitive processing (Cohn et al., 2018; Barach et al., 2021; Scheffler et al., 2022; Kaye et al., 2023) by means of methodologies borrowed from language processing and originally developed for words like self-paced reading, eye tracking, neurolinguistic imaging, or acceptability rating studies. It is known that the processing of verbal material can be influenced by a variety of stimulus-related factors like various affective (e.g., valence and arousal; see Pratto & John, 1991; Kousta et al., 2009; Kuperman et al., 2014), semantic (e.g., concreteness, imagability; Barber et al., 2013), form-based (e.g., word length, stress pattern, grammatical gender (Barton et al., 2014)), and other (e.g., word frequency, age of acquisition; Brysbaert et al., 2018; Johnston & Barry, 2006) dimensions. In order to control for these factors, they have been studied extensively and form large publicly available databases of psycholinguistic measures for linguistic expressions, which can inform further research. For single words, established databases present norms on affective and psycholinguistic dimensions (Coltheart, 1981; Warriner et al., 2013; Winter et al., 2023). There is some effort to link databases across multiple studies and languages (Tjuka et al., 2022). In addition, there are already some norms for multi-word expressions like idioms and proverbs (Benjafield et al., 1993; Bonin et al., 2018; Citron et al., 2020; Müller et al., 2022; Muraki et al., 2023).

Beyond the research on cognitive linguistic processing, ratings of verbal material can be beneficial in many ways. According to Warriner et al. (2013), affective ratings of words are also useful for research on affect itself, estimation of the sentiment of a complete utterance based on the values of its single words, and automatic estimation of the affective values of new words based on previously known ratings. In studies with vulnerable populations such as mental health patients, elderly persons, or language learners, certain types of stimulus material exhibit specific patterns of processing (Garcia-Leon et al., 2021; Kauschke et al., 2019; Kensinger et al., 2002; Ponari et al., 2018; Reed & Carstensen, 2012). In addition, word norms on emotions and complexity can yield important information in text construction, for example in order to implement a certain style or adhere to a maximum reading level.

Currently, emoji research in linguistics and psychology is hindered by a lack of standardized evaluations of emojis’ affective and interpretive ratings, which prevents many types of comparisons across emojis or items. So it only seems plausible that these properties should also be studied in relation to emojis. In addition to the research fields described by Warriner et al., emoji ratings can contribute to the understanding of emoji conventionalization over time. Since emojis are used in various linguistic contexts, understanding their interpretation and processing may also help to answer the question of whether emojis have ubiquitous transcultural and translinguistic meanings, or rather show culturally specific uses and characteristics.

In current psycholinguistic studies using emojis, their meaning and lexical properties are often determined ad hoc by the experimenters (e.g., Wood & Ruder, 2016; Weissman & Tanner, 2018; Tang et al., 2020; Scheffler et al., 2022). Alternatively, Emojipedia or Unicode definitions may be used (Barach et al., 2021). However, these definitions are often vague or unspecific and do not capture the dynamics of meaning evolution or lexicalization. It is known that many existing emojis are ambiguous (Częstochowska et al., 2022) or can be interpreted in various ways (Miller et al., 2016). The Unicode consortium currently even requires new face emojis to be ambiguous in order to be considered for introduction.^1^ Often, emojis also take on new meanings or connotations over time (examples are the  as a phallic symbol and the “slightly smiling”  emoji, which has been reinterpreted as indicating “I’m dead inside”, particularly by younger users). In order to provide a solid foundation for further linguistic and psycholinguistic studies using stimuli with emojis, it is necessary to establish affective and semantic norms for them similarly to the existing resources for words in many languages. In this paper, we contribute to this effort by introducing the, to date, most comprehensive norming study of face emojis conducted with German speakers.

As of Unicode version 15.0, there are 3664 available emojis.^2^ These cannot be considered a uniform class. There is accumulating evidence that there are at least two distinct types of emojis that can be distinguished both in theory and through observation: face emojis and activity emojis. These two types of emojis exhibit unique linguistic characteristics (Kaiser & Grosz, 2021; Maier, 2023). Interestingly, face emojis, although much fewer in quantity compared to the rest of the emojis, are much more widely used (Ferré et al., 2023). In one study by the Unicode foundation from 2021, just 100 emojis make up 82% of all emoji uses^3^; many of them are face emojis. In consequence, face emojis have been the focus of a large part of the research on the linguistics of emojis.

To date, only a few studies have provided subjective norms for emojis and to our knowledge there is just one very small study on the matter conducted with a German-speaking population. For English, to our knowledge, only valence and arousal have been tested before. We list the available resources in Table 1.

**Table 1:** Human rating studies for subjective evaluation and descriptive meaning of emojis

Study	Year	Language	No. of Face Emojis	No. of total Emojis	Arousal	Valence	Clarity	Visual Compl.	Familiarity	Other	Meaning	Comment
Kralj Novak et al.	2015	Crosslinguistic	57	751	(x)	(x)						In-context evaluation (entire tweets)
Rodrigues et al.	2018	Portuguese	76	78	x	x	x	x	x	Meaningfulness	1 free text	Different platforms
Jaeger et al.	2019	English (USA)	33	33	x	x					1 free text	("Mood/feeling")
Jones et al.	2020	English (USA)	70	70		x			x			
Fischer and Herbert	2021	German	18	18	x	x						Compared to human faces, emoticons
Was and Hamrick	2021	English (USA)	90	105	x						1 free text	
Kutsuzawa et al.	2022	Japanese	74	74	x	x						
Ferré et al.	2022	Spanish	99	1031	x	x	x	x	x	Subjective frequency ratings		
Częstochowska et al.	2022	English (USA)	107	1289							1 free text	
Li & Wang	2022	Chinese	37	90	(x)	(x)						Weibo emojis

“(x)” indicates that a comparable, but not identical category was tested (e.g., sentiment instead of valence)

Rodrigues et al. (2018) were the first to produce a database of subjective ratings for 153 common emojis from different platforms (77 from iOS, 63 from Android, nine from Facebook, and four from Emojipedia); these include 76 face emojis. In particular, they examined aesthetic appeal, familiarity, visual complexity, clarity, valence, arousal, and meaningfulness, with Portuguese speakers. The authors showed that emojis surpass emoticons in terms of familiarity, arousal, positive valence, and clarity of meaning. Gender distinctions in emoji ratings were apparent, with women attributing higher familiarity, clarity, and meaningfulness to emojis compared to men. Interestingly, variations were observed in the evaluations of iOS and Android emojis, with iOS emojis receiving higher ratings for familiarity, clarity, and meaningfulness. No distinctions emerged in the fundamental affective components of arousal and valence. In addition to this, the authors report that the majority of dimensions are highly correlated. Since then, several studies have focused on facial emojis and examined their affective or semantic properties by means of subjective ratings of US English speakers, as well: Jaeger et al. (2019) collected both valence and arousal ratings and meanings via an open-ended questionnaire for 33 face emojis. In their study, they focus on the use of emojis in consumer research and demonstrate the U-shaped relationship between valence and arousal, indicating that more positive or negative emojis were also more arousing. Jones et al. (2020) investigated gender-related differences in valence and familiarity ratings for 70 facial emojis, and found a bias for negative emojis in female participants; and Was & Hamrick (2021) collected one free-text emoji meaning and arousal ratings for 105 emojis, including 90 face emojis. In a more recent study with German speakers, Fischer & Herbert (2021) compared ratings for arousal and valence for 18 emojis with ratings for emoticons and faces with a female-biased sample. Fischer & Herbert report that emojis and faces are rated with a similar emotionality. Ferré et al. (2023) constructed the largest database to date of 1031 emojis rated for visual complexity, familiarity, frequency of use, clarity, emotional valence and arousal, based on ratings by Spanish native speakers. The authors replicated the U-shaped curve between valence and arousal. Furthermore, they found that the relationship between familiarity and valence (positive emojis were also rated as more familiar) mirrors the same correlation in words and concluded that emojis are suitable stimuli to study emotions. Facial emojis and people emojis were found to be used more frequently and were more familiar compared to other categories such as activity emojis and symbols. Overall, subjective ratings of familiarity serve as a reliable indicator of emoji usage. Ferré et al.'s results also demonstrated medium-to-high correlations with previous rating studies, with agreement between participants being higher for valence than for arousal.

There are also a few studies with non-Western participants. Kutsuzawa et al. (2022) clustered 74 facial emojis based on their valence and arousal ratings by Japanese speakers, identifying six clusters of emojis corresponding to distinct emotional states. Li and Wang (2022) asked Chinese users to rate 52 Weibo emojis, including 37 faces, on (categorical) sentiment and intensity (corresponding to arousal). Their study focused on the sentiment and intensity of emoji usage in evaluating public opinions on social media. They reported that the sentiment derived from emoji usage reflects the general public attitude toward an event. In cases where there is a positive attitude towards an event, the number of emojis with positive valence will outnumber the negative emojis; however, notably, the intensity of the negative emojis was consistently higher compared to the positive emojis. The authors concluded that the frequency and intensity of positive and negative emojis follow distinct patterns in the discussion of controversial events.

In addition to direct ratings by human participants, several studies aim at grasping the affective and semantic characteristics of emojis based on their naturalistic use in context. Kralj Novak et al. (2015) compute numerical scores for sentiment (~ valence) and neutrality (the complement of arousal) for 751 emojis (including 57 face emojis) based on categorical ratings of 1.6 million tweets across 13 European languages. They use this to derive composite sentiment scores for emojis, which have been used in sentiment analysis approaches.

Emoji meaning descriptions have been considerably less studied. In one main exception, Częstochowska et al. (2022) calculated ambiguity scores for 1289 emojis based on one-word meaning descriptions in English by US participants. They reported a significant variation in ambiguity scores, ranging from emojis that are completely unambiguous to those whose meaning is extremely variable. Face emojis fall in the middle of the ambiguity scale, being more ambiguous compared to objects, activities, or clothing and accessories. Weissman (2022) has also explored emoji meanings using single English descriptions in a pretest for an experiment, but not including face emojis. Much earlier, Barbieri et al. (2016) have automatically computed dense distributional vector representations (so-called embeddings) for emojis based on their occurrence in a large tweet corpus. By analyzing emojis identically to word tokens, this distributional approach enables a direct comparison of emojis to words, for example by way of calculating an emoji’s most similar word neighbors in the vector space (e.g.,  resembles *love*, *babe*, *youu*, *awww*, *bby* in their study). In contrast, Eisner et al. (2016) computed emoji embeddings from the descriptions of all Unicode emojis. These embeddings can be used in the same way as the usage-based ones, but encode the “dictionary” descriptions of the emojis’ meaning instead of their usage-based meaning derived from a corpus.

Based on the existing (partial) ratings for certain emojis, specifically for valence and arousal, it is possible to investigate whether correlation effects known for lexical processing also manifest in emoji processing. For example, Kaye et al. (2021, 2023) explored the effect of emotional valence ratings from Rodrigues et al. (2018) on human participants’ accuracy in a lexical decision task. They report a null effect. In a study on reading times including stimuli with emojis, Scheffler et al. (2022) conjecture that ambiguity and visual complexity of (object) emojis has an effect on their processing time during reading, manifesting in between-item differences. Weissman et al. (2023) revealed an interesting trend: emojis with higher rates of meaning agreement tended to elicit quicker reaction times in a match/mismatch task. Other experiments using emoji stimuli do not specifically control for these possibly confounding variables, in part because reliable norms have not been available. In the current study, we aim to address this gap.

In order to provide a solid base for future emoji research, our study combines norms for valence and arousal expressed by emojis (which have been the focus of previous analyses because of face emojis’ natural connection with human emotions) with less-studied lexical properties of emojis that may influence the results of psycholinguistic experiments including emoji stimuli. These properties include the visual complexity of an emoji, its familiarity, and its clarity. All of these characteristics may influence an emoji’s processing time and impact the interpretation of utterances with emojis.

We additionally included a description task in our survey. The results of this task yield new, normed senses for all 107 face emojis based on German-speaking participants. While existing listings such as Emojipedia provide emoji meanings, these definitions are often incomplete and do not reflect the true ambiguity and current usage of emojis. Our descriptive norms allow us to derive context-free meanings for all face emojis. Finally, we also provide corpus frequencies from public (Twitter) and private (WhatsApp) digital interactions for all 107 existing face emojis.

## Methods

### Participants

Thirty-eight undergraduate students participated as part of a linguistics class taught by the first author. An additional 115 German-speaking participants from Germany were recruited via the platform Prolific. All participants completed the online questionnaire after reading and accepting an informed consent form. The sample size was selected based on similar studies so that each stimulus is rated by at least 30 participants. Based on predefined criteria, we excluded 15 participants who stated a first language other than German. The remaining 138 participants (60 female, 78 male) had an average age of 32.3 years (SD = 10.7, range, 18–70 years). The participants from Prolific received £3.40 (on average, £13 per hour) for their participation, the students received partial course credit.

After completing the stimulus questionnaire, the participants were shown a short demographic survey, including questions about their social media habits and emoji familiarity. Almost 90% of the participants indicated frequent use of emojis in computer-mediated communication (“almost always” 32.6%, “often” 34.1%, “sometimes” 22.5%). Only 10.1% self-rated their use as infrequent (“rarely”) and one person (0.7%) stated that they “never” use emojis. As in previous studies, female participants use emojis more frequently than male participants (see Appendix). Over 94.1% of the participants stated that they use WhatsApp on a regular basis (“multiple times per day” 54.3%, “daily” 20.3%, “every few days” 14.5%, “once in a while” 4.6%), the remaining 6.5% never used the messenger. 68.6% use Android and 29.2% use iOS as operating systems.

### Stimulus set

In our study, we focus on the 107 human face emojis up to the “clown face” in the Unicode list. This includes all facial emojis that were available at the time of data collection. All stimuli were downloaded from Emojipedia and included as images. As characters, emojis are defined in the Unicode description. However, each platform may implement their own specific emoji designs. Our study aims at the interpretation of emojis by the German-speaking population. We assume that most people encounter (and are able to understand) different types of emoji designs in their daily life (e.g., Apple’s  vs. WhatsApp’s  ): on their devices, on TV or videos, on printed media or even other common objects. Thus, in contrast to some previous studies (notably, Rodrigues et al., 2018), we only use one set of emoji stimuli. Given that most emojis are used in personal smartphone chats and by far the most prevalent chat platform in Germany is WhatsApp, we opted to use WhatsApp emoji designs for the study. The current WhatsApp designs additionally are in most cases very similar to the Apple designs (which are shown to iOS users on all chat apps).^4^ In order to control for any remaining effects of differences in the emoji design, we present the stimuli as images, using only the most widespread design in Germany, WhatsApp emojis.

### Procedure and measures

**Fig. 1:**
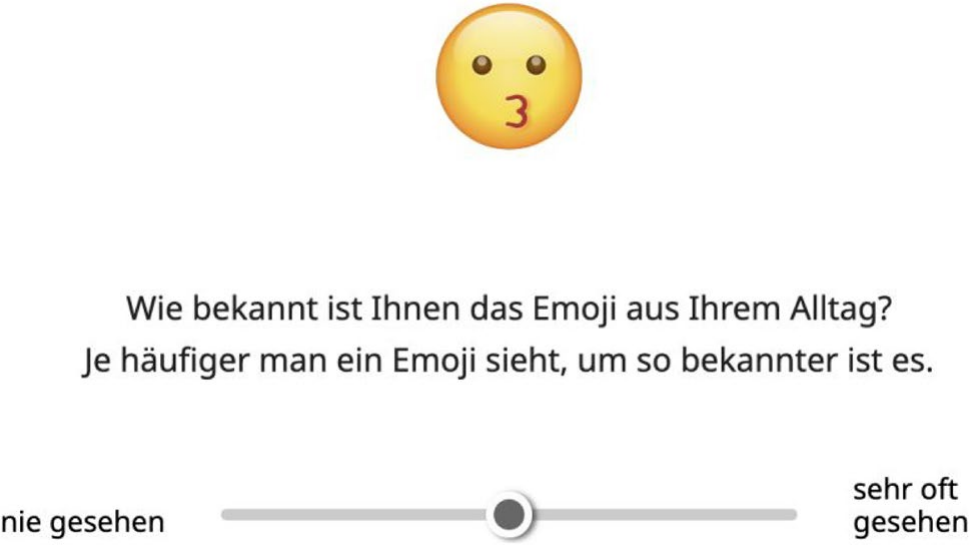
Sample familiarity rating screenshot. The task beneath the emoji states “How familiar is the emoji from your daily life? The more often you see an emoji, the more familiar it is.” The scale on the slider ranges from “never seen” to “seen very often”

The set of 107 emojis was split into five subsets of 21 or 22 emojis, each participant was randomly assigned to a group and shown one emoji subset. The questionnaire consisted of six sections, which each focused on only one measure. Each section started with an instruction screen, after which the individual emojis from the subset were presented in random order. The participants rated familiarity, visual complexity, clarity, valence, and arousal by moving a slider on a continuous scale. Subsequently, the scale was internally calculated to span from 0 to 100. One example rating screen is shown in Fig. 1. Additionally, the participants were asked to name up to three meanings for each emoji, via free text fields.

In order to avoid any effects of habituation, “familiarity” was the first section for evaluation. We adopted a common conceptualization of familiarity, namely the subjective frequency of exposure to a single emoji (Citron et al., 2016; Rodrigues et al., 2018; Ferré et al., 2023). The extremes on the sliding scale were labeled “never seen” and “seen very frequently”.

After the familiarity section, participants answered the meaning description questions. This order was chosen to avoid influencing the meaning responses by the following semantic rating sections. Participants were asked to name the meaning of each emoji using up to three phrases, for which they had three separate text fields. After this naming task, the participants completed the remaining ratings in the order listed here.

Visual complexity describes the visual properties of the emoji in terms of details. The extremes on the rating slider were labeled “very simple” and “very complex”.

Clarity relates to the number of different meanings an emoji usually conveys. Emojis representing a single meaning are clear and unambiguous in contrast to emojis representing more than one meaning, which are ambiguous or vague. The rating scale ranged from “very clear and unambiguous” to “very unclear or ambiguous”. “Clarity” was chosen as the measure because it is easy to capture intuitively. In contrast, there may be many reasons why an emoji’s meaning is unclear, among them ambiguity, vagueness, lack of (cultural) understanding, processing errors, etc.

Furthermore, we measured the core affective properties of the emojis (Feldman Barrett & Russell, 1998; Russell, 2003). Emotional valence represents the extent to which an emoji is perceived as positive/pleasurable vs. negative/displeasurable. The rating scale ranged from “very negative” to “very positive”. Emotional arousal represents the physiological activation on the axis “very calm” to “very arousing”. Both valence and arousal have been operationalized similarly in previous emoji norming studies (Rodrigues et al., 2018; Jaeger et al., 2019; Kutsuzawa et al., 2022; Ferré et al., 2023).^5^

After completing the ratings and the naming task, the participants were asked basic demographic questions (age, gender, level of education) and stated the frequency of their own emoji use and the operating system of their smartphones.

### Face emoji frequency

While it is known that face emojis are among the most used emojis, and generally, emojis are encountered frequently in digitally mediated text, it is hard to come by exact figures for specific emojis’ frequencies of use. Even the Unicode consortium reports only a ranking of emojis by frequency, using data “from multiple sources”, but without providing exact numbers.^6^ Additionally, these ranks are global and not tailored to a specific region or language. In order to better validate the subjective ratings of the emojis included in our survey, we also report frequency norms for each emoji. We report two kinds of face emoji frequencies in German data: first, frequencies in public data, i.e., a large German-language tweet corpus; and second, frequencies in German private WhatsApp chats. Both data sets complement each other as the chat data are necessarily much smaller than the Twitter data and does not contain all emojis in sufficient quantity for statistical analysis.

**Fig. 2:**
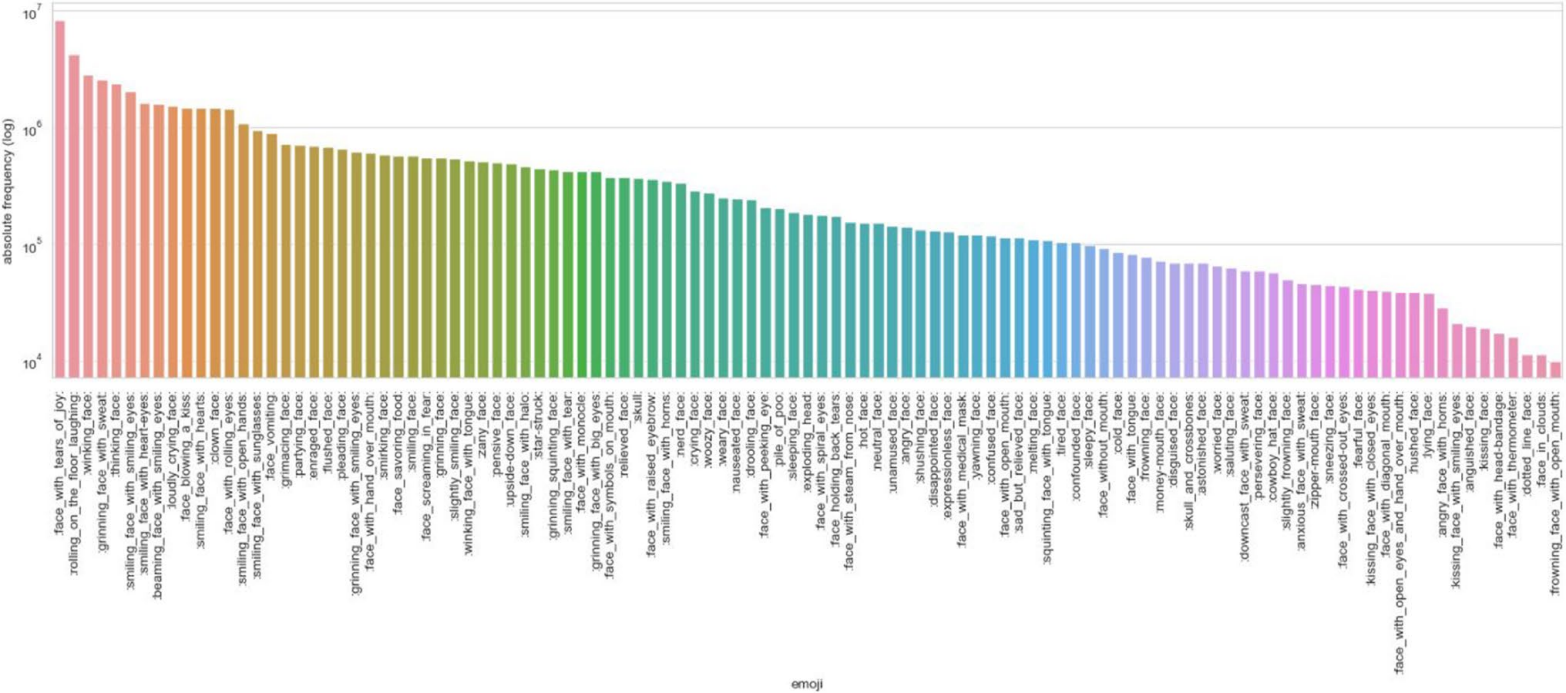
Face emoji frequency (log scale) in a large corpus of German tweets (2022)

The public face emoji frequencies are based on a large corpus of German-language tweets, which were collected using the method proposed in Scheffler (2014), by searching for a list of high-frequency German function words in real time. We used the entire collection of over 280 million German tweets sent during the year 2022, almost 800,000 tweets per day. This data set represents the majority of all German tweets composed during this period, the last year for which relatively complete data was available. The year 2022 was chosen because several of the face emojis we tested were only approved at the end of 2021 and only introduced to most platforms during the course of 2022. It is worthwhile to note that a few of these new emojis, for example “face with peeking eye”  , are nevertheless used relatively frequently. Each face emoji was counted in this corpus, counting duplicate occurrences separately. The face emojis’ absolute frequency ranged from just under 10,000 (“frowning face with open mouth”  ) to over 8.3 million (“face with tears of joy”  ), and is shown in Fig. 2.^7^

In addition, we searched for all 107 face emojis in the only publicly available WhatsApp corpus containing recent German data, the MoCoDa2 database.^8^ At the time of writing, the corpus contains over 300,000 tokens in 1001 chats. 101 of the face emojis were found in the corpus, with frequencies between 1 (three emojis) and 2433 (“face with tears of joy”  ). Forty emojis occur fewer than ten times. An emoji’s frequency in WhatsApp is, however, strongly positively correlated with its frequency in the huge Twitter corpus (*r =* .782), so that the much larger and more robust Twitter frequencies can be studied further.

## Rating results

### Reliability and validity

#### Interrater reliability

To evaluate the interrater reliability, we measured the intraclass correlation coefficient using the *pingouin* Python package (Vallat, 2018) to calculate the two-way random effects based on the absolute agreement of multiple raters (Koo & Li, 2016). All ratings showed high reliability (valence ICC2k > .98, CI [.97, .98]; *p* < .001, arousal ICC2k > .90, CI [.88, .95], *p* < .001; familiarity ICC2k > .91, CI [.89, .97], *p* < .001, clarity ICC2k > .90, CI [.87, .97], *p* < .001; visual complexity ICC2k > .95, CI [.93, .98], *p* < .001).^9^

#### Validity

To estimate the validity of our ratings, we compared our mean scores with the rating means reported in the other recent rating study. For normally distributed ratings, we used the Pearson correlation coefficient. The majority of ratings demonstrated significant, positive correlations. Our arousal ratings exhibited a high positive correlation with the findings of Ferre et al. (*r* = .824, *p* < .0001), a medium positive correlation with those of Rodrigues et al. (*r* = .62, *p* < .0001), and a low correlation with the data from Kutsuzawa et al. (*r* = .27, *p* < .05). The correlation with the ratings from Jaeger et al. was nonsignificant. Additionally, our familiarity ratings showed medium to high positive correlations with previous studies: Rodrigues et al. (*r* = .72, *p* < .0001), Ferré et al. (*r* = .69, *p* < .0001), and Jones et al. (*r* = .78, *p* < .0001). Similarly, our clarity ratings were positively correlated with previous studies (Rodrigues et al. *r* = .55, *p* < .0001; Ferré et al. *r* = .49, *p* < .0001). Furthermore, our ratings for visual complexity exhibited significant positive correlations with results from previous studies, including Rodrigues et al. (*r* = .65, *p* < .0001) and Ferré et al. (*r* = .62, *p* < .0001). As valence follows a non-normal distribution, we employed Spearman rank correlation. All correlations between our data and previous studies were highly positive: Jaeger et al. (*r* = .91, *p* < .0001); Rodrigues et al. (*r* = .91, *p* = .0001); Ferré et al. (*r* = .91, *p* < .0001); Was et al. (*r* = .94, *p* < .0001); Jones et al. (*r* = .93, *p* < .0001); Kutsuzawa et al. (*r* = .91, *p* < .0001).

#### Descriptive results

The descriptive statistics of the results are shown in Table 2. The face emojis have a medium visual complexity and appear to have above average familiarity and clarity. They have a medium arousal and valence. Figures in the Appendix show the individual subjective ratings for each emoji.

**Table 2:** Descriptive statistics of the emoji ratings

	Mean	SD	Min	25%	50%	75%	Max
Complexity	47.25	27.19	0.0	25.0	50.0	68.0	100.0
Familiarity	61.53	32.56	0.0	34.0	67.0	91.0	100.0
Arousal	55.18	27.30	0.0	34.0	60.0	75.0	100.0
Clarity	59.54	31.61	0.0	32.0	66.0	88.0	100.0
Valence	48.08	30.61	0.0	24.0	44.0	74.0	100.0

**Fig. 3:**
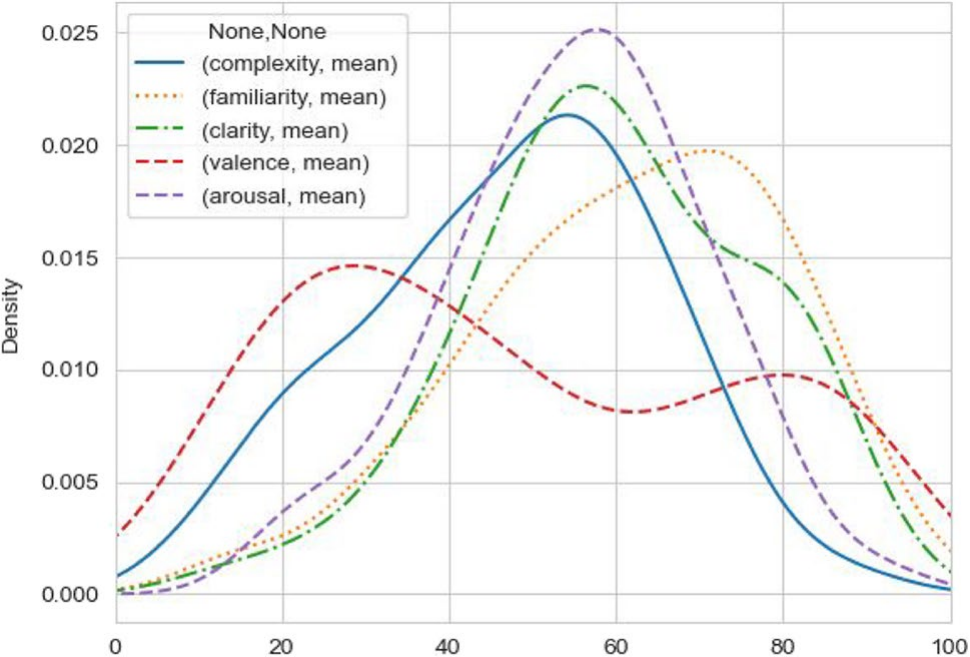
Distribution of ratings for all measures

The ratings show that the set of face emojis covers the entire range of the scale for each measure, indicating the large variability of emojis as a category. The density plot in Fig. 3 demonstrates the distributions of the ratings for the different measures. All measures except valence follow an approximately Gaussian distribution, while valence is bi-modal.

#### Correlations between different measures

In this section, we report the correlations between the measured variables. For variables with normal or nearly normal distribution (arousal, familiarity, visual complexity, clarity), we calculated Pearson correlations. Since valence shows a bimodal distribution and frequency an exponential one, we report Spearman rank correlations for these measures. The significance level was set at *p* < .05. The statistical analysis was done with the Python package SciPy (Virtanen et al., 2020).

#### Familiarity and frequency

**Fig. 4:**
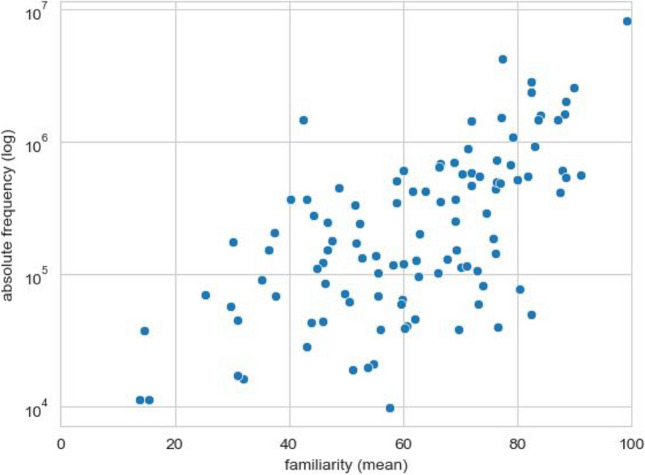
Correlation of subjective familiarity and absolute frequency (Twitter)

The familiarity measure captures the subjective frequency of exposure to each emoji (Citron et al., 2016; Rodrigues et al., 2018; Ferré et al., 2023). Figure 4 compares the mean familiarity rating of the emojis studied with their objective, absolute frequency of occurrence (log scaled) in the large corpus of German tweets discussed above. Both measures show a substantial correlation (Spearman rank correlation *r* = .62, *p* < .0001). The familiarity ratings are even more strongly correlated with the WhatsApp frequencies of the emojis (*r* = .80, *p* < .0001).

#### Affective ratings

Furthermore, we examined the linear correlations of all affective and semantic variables, as well as the frequency of occurrence, presented in Table 3. We found highly significant positive correlations for familiarity and valence (*r =* .39, *p* < .001) and for familiarity and clarity (*r =* .50, *p* < .001). Visual complexity showed a highly significant negative correlation with familiarity (*r =* – .42, *p* < .001) and with clarity (*r =* – .27, *p* < .01) and a weak positive correlation with arousal (*r =* .25, *p* < .01). Valence and arousal showed a significant negative correlation (*r =* – .56, *p* < .001). Frequency is positively correlated with valence (*r =* .43, *p* < .001) and familiarity (*r =* .62, *p* < .001). All other correlations are not significant.

**Table 3:** Correlations among subjective measures, as well as their correlation with objective frequency

	Arousal	Valence	Clarity	Familiarity	Complexity
Valence	– .56 ***				
Clarity	.16	.05			
Familiarity	– .11	.39 ***	.50 ***		
Complexity	.25 **	– .07	– .27 **	– .42 ***	
Frequency	– .02	.43 **	.14	.62 ***	.01

^*^ *p* < .05, ** *p* < .01, *** *p* < .001

**Fig. 5:**
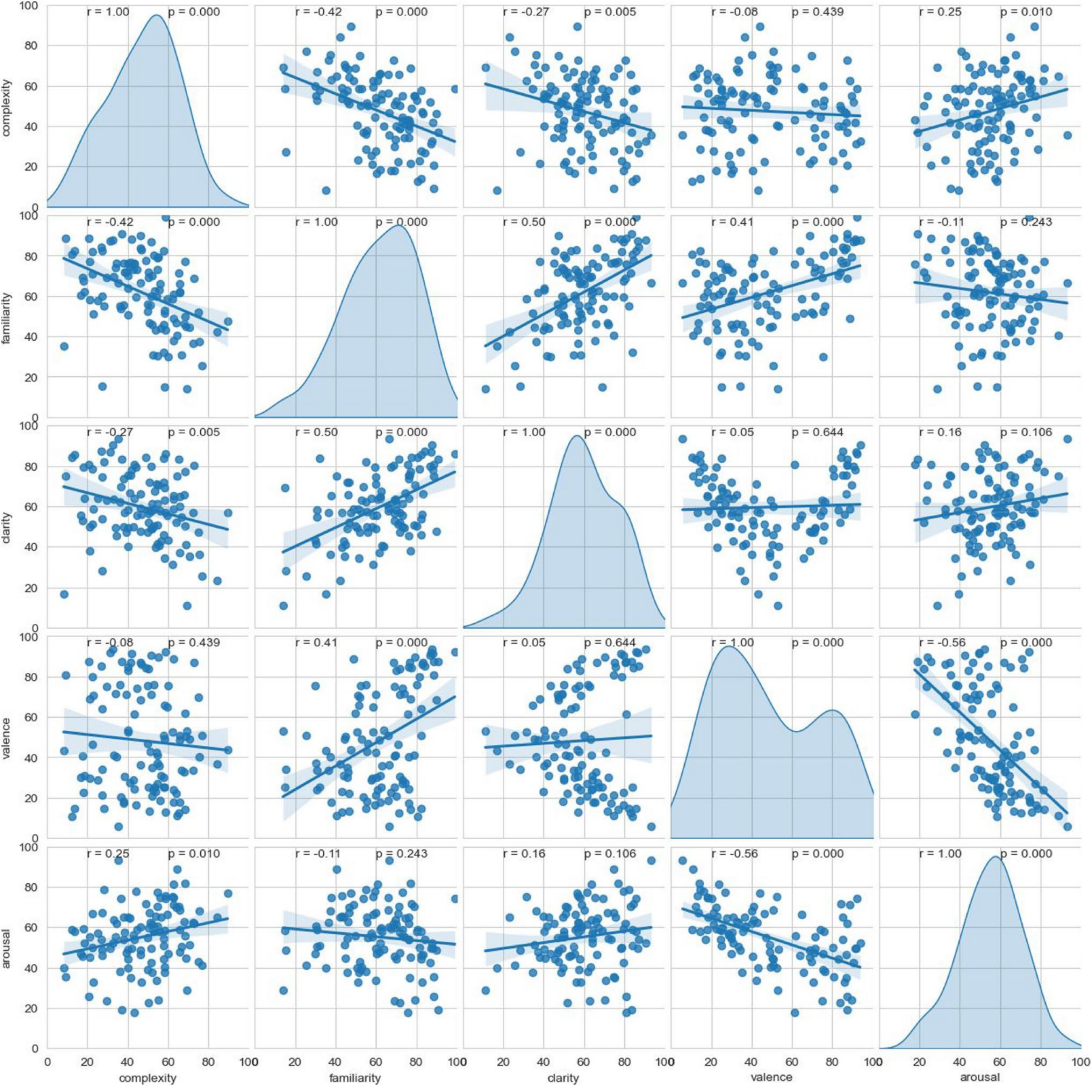
Correlations between all rating measures. The diagonals show kernel density estimates (KDE) for each variable, which represent the distribution of the data using a continuous probability density curve

Pairwise linear relationships between the measures are depicted in Fig. 5.

**Fig. 6:**
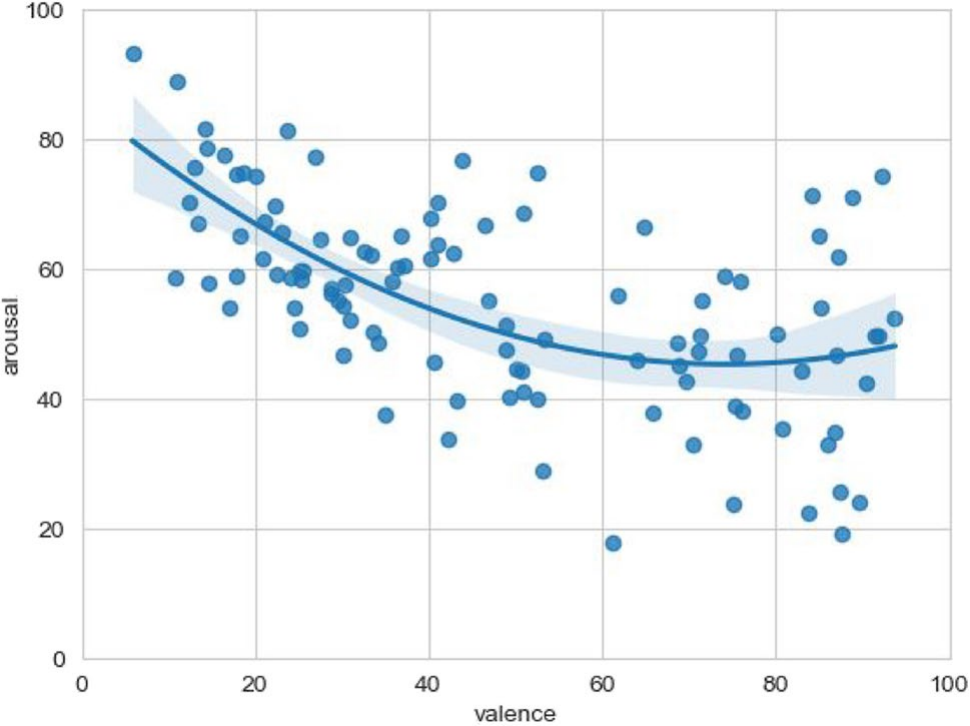
Mean arousal and valence of the face emojis

The correlation between the two affective measures arousal and valence is of particular interest. We observed that a quadratic trend model, which yielded an R-squared value of .38 (F(2, 103) = 31.34, *p* < .001), is a better fit for the data compared to a linear trend model (R-squared value of .31, F(1, 104) = 46.94, *p* < .001). High arousal corresponds to either a high or low valence, while medium valence corresponds to low arousal, resulting in a U-shaped curve (Fig. 6).

#### Emoji meaning: Survey results and analysis

In addition to the affective and semantic ratings, we also elicited up to three free text meaning descriptions for each emoji from each participant. Emojis are defined in the Unicode code book, but their use and interpretation in practice diverges wildly from those listed senses (see e.g., Miller et al., 2017; Gawne & McCulloch, 2019; Weissman, 2019). Previous work has allowed only one free form meaning for each emoji (Rodrigues et al., 2018; Jaeger et al., 2019; Was & Hamrick, 2021; Częstochowska et al., 2022), but we believe that the emojis’ ambiguity is better captured by allowing each participant to enter several different senses. In this section, we report the result of the emoji meaning task, where we asked participants to type up to three “meaning descriptions” (*Benennungen*) for each face emoji. Each emoji received an average of 58 individual descriptions (min: 47, max: 70).

Table 4. shows the elicited senses for four sample emojis, normalized for spelling and translated into English. It can be seen that the list of senses is very diverse, though specific trends emerge. The most common emoji “face with tears of joy”  has only one main meaning, ‘funny/amusement’ or ‘laughing’. It is also an emoji for which most participants only specified one or two descriptions (47 in total). In contrast, the “slightly smiling face”  , which was described by one participant as “just the normal smiley”, has clearly distinct senses. In addition to the predominant meaning of ‘happiness/joy’, participants also listed it as marking ‘passive aggressive’ moves and for some other related uses. It is also interesting to look at two visually similar emojis, the “angry/smiling face with horns”  and  , which differ only in the mouth shape. Even though ‘evil’ is named by far the most often for both emojis, subtle meaning differences can be discerned in the other senses: While the frowning face  is understood as ‘angry’ or ‘in a bad mood’, the evilness of the smiling face  has a ‘cheeky’ component, described as ‘Schadenfreude’, ‘teasing’, ‘tricky’ or ‘sexy’.

**Table 4.** Meaning descriptions for four sample emojis

Face with tears of joy	Slightly smiling face
9 laugh, 8 funny, 2 very funny, 2 laugh tears, 2 extremely funny, 2 amused, 1 you laugh, 1 violent laugh, 1 very joyful, 1 sounding laugh, 1 something is very funny, 1 positive, 1 make fun of something or someone, 1 lol, 1 laughing tears, 1 laughing fit smiley, 1 laughing fit, 1 laughing, 1 laugh until you cry, 1 laugh at someone, 1 joy, 1 joking, 1 highest form of amusement, 1 happy, 1 haha, 1 find something very funny, 1 excessive laughter, 1 big amusement	7 happy, 6 joy, 5 smile, 5 friendly, 4 positive, 3 nice, 2 smiley, 2 satisfaction, 2 passive aggressive, 2 cheerfulness, 1 unsure be which emoji otherwise should be used, 1 satisfied, 1 sarcastically meant smile, while you fall completely from faith internally., 1 relaxed, 1 pretend that you mean something nice, 1 passive aggressive smile, 1 normal, 1 nice smile, 1 neutral, 1 like, 1 laugh, 1 just the normal smiley, 1 in a happy mood, 1 in a good mood, 1 harmless, 1 good-humored, 1 fun, 1 everything good, 1 everything cool, 1 encouraging, 1 easy, 1 completely overwhelmed in a situation, which is why you simply freeze (see: It's fine meme)., 1 attentive, 1 answer to show that you have read it, 1 agreement, 1 I like it
Angry face with horns	Smiling face with horns
14 evil, 6 anger, 5 angry, 4 devilish, 3 mad, 2 irate, 2 devil, 1 very annoyed, 1 very angry, 1 that's mean, 1 that's evil, 1 stubborn, 1 strong anger, 1 someone is mad, 1 someone is angry, 1 revenge, 1 naughty, 1 maximally increased anger, 1 hate, 1 evil action, 1 devil , 1 death, 1 consciously evil for fun, 1 caution, 1 be mad, 1 bad mood, 1 I'm angry, 1 I will bring the means of production back into the hands of the workers - kind of vibe, 1 I go berserk, 1 I am stinking mad, 1 I am angry	12 evil, 7 devil, 4 devilish, 3 deceitful, 3 cheeky, 2 sneaky, 2 sexy, 2 Schadenfreude, 1 you tease someone, 1 strength, 1 spitish, 1 spicy, 1 self-confident, 1 said something mean, 1 rate something as evil, 1 plan something, 1 plan, 1 mischievous, 1 mean, 1 made a dirty joke, 1 look forward to something, 1 little devil, 1 ironic, 1 insidious, 1 horny, 1 fun with tricks, 1 dirty, 1 devilish plan, 1 devilish (in the funny sense), 1 demon, 1 cunning, 1 coolness

Meaning descriptions for four sample emojis (translated by the authors), the numbers preceding each sense show how often this description was listed by participants

### Preprocessing

The free text entries for the senses vary a lot in spelling and form, some even include punctuation. In order to enable further analysis, we preprocessed the descriptions with the spaCy model for German de_core_news_lg.^10^ We tokenized and lemmatized all sense entries, and removed stop words, to produce a vocabulary of content words characterizing each emoji. Words occurring more than once were retained in this list as many times as they occurred, and multi-word expressions were split into their individual tokens.

#### Correlations with subjective ratings

In order to quantitatively assess the sense descriptions, we extracted measures of semantic variation, arousal, and valence from the text data and correlated them with the subjective ratings obtained in the first part of this study.

**Fig. 7:**
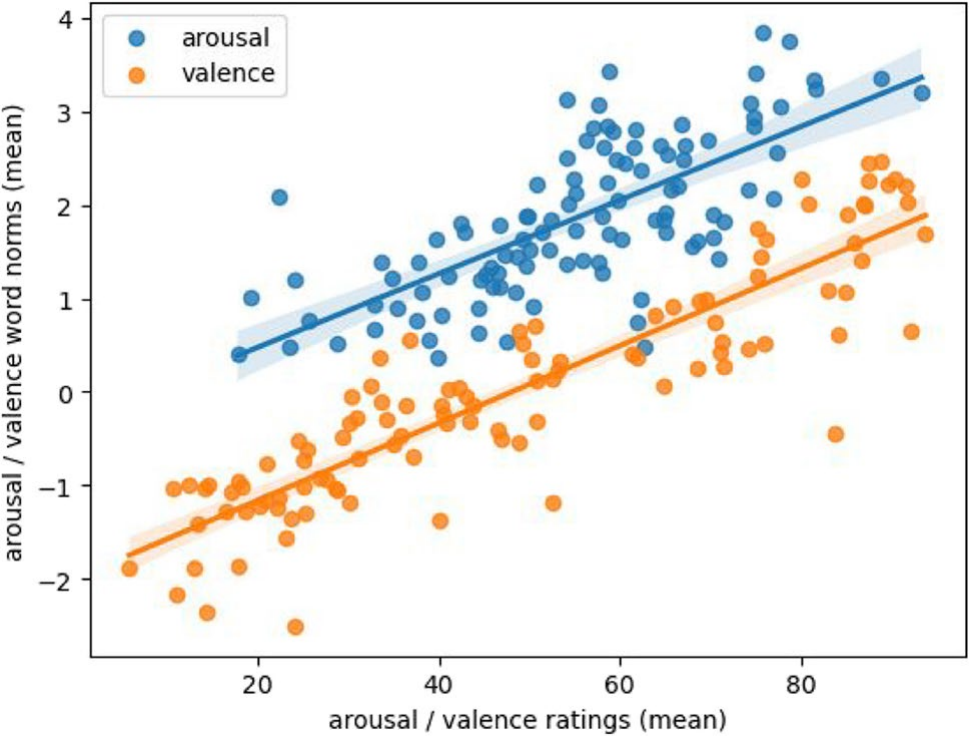
Subjective arousal and valence ratings of emojis compared with arousal and valence words norms of the emojis’ semantic descriptions

First, we calculated description-based valence and arousal values for each emoji based on valence and arousal norms for the individual words used in the descriptions. We used the word norms from the newly developed GLEAN dataset for German (Lüdtke & Hugentobler, 2022). The mean emoji valence from our participants’ subjective ratings and the average valence for the vocabulary of that emoji’s descriptions showed a highly significant strong correlation (Spearman *r* = .90, *p* < .0001). Similarly, the mean emoji arousal from the subjective ratings and the average arousal of the words used to describe the emoji also showed a highly significant strong correlation (Pearson *r* = .72, *p* < .0001). Both correlations are depicted in Fig. 7.

**Fig. 8:**
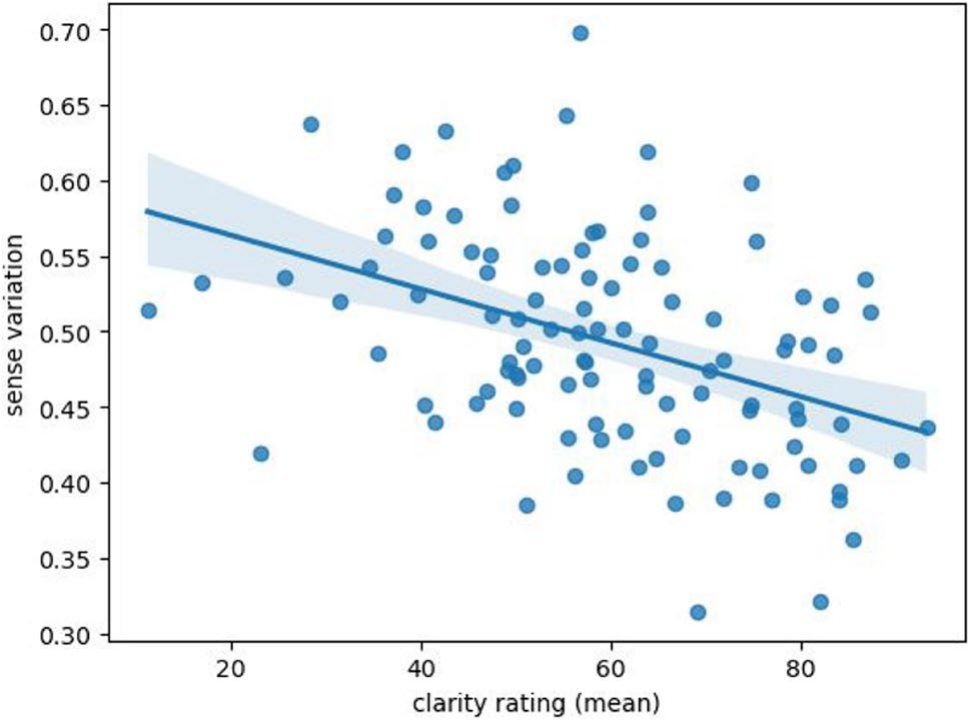
Correlation between the mean clarity rating and the sense variation for each emoji

Second, we calculated the “semantic variation” of the emoji sense descriptions and compared this value to the emojis’ clarity rating, since an emoji should be rated less unambiguous if it has more diverse meaning descriptions. The semantic variation was calculated with the procedure proposed by Częstochowska et al. (2022). They use word embeddings (Mikolov et al., 2013) to represent the meaning of each word describing an emoji using high-dimensional numerical vectors. These embedding vectors represent each word (or emoji) in a 300-dimensional space, so that words with similar meanings are located closer to each other than less similar words. Closeness in a vector space is often estimated using the cosine measure. For example, the vector for ‘cat’ would have a smaller cosine distance from the vector embedding for ‘kitten’ than from the vector for ‘jellyfish’. This kind of meaning representation makes it possible to distinguish between similar meaning descriptions (e.g., ‘devilish’ and ‘devil’ from Table 4.) versus less similar meaning descriptions (e.g., ‘evil’ and ‘anger’ from Table 4.), in a principled way. Częstochowska et al. (2022) define the “semantic variation” of an emoji as the weighted cosine distance between each word used for an emoji and that emoji’s modal (= most common) meaning. For the emojis in our study, the semantic variation shows a highly significant medium correlation with the mean of the clarity ratings (*r* = – .42, *p* < .0001) (see Fig. 8).

#### Distributional semantics of emojis

In order to further analyze the meaning descriptions of the face emojis in our study, we provide distributional representations (so-called word embeddings) for them. Vector embeddings for emojis should be compatible with vector embeddings for the regular words of a language by locating emojis in the same space as words. This makes it possible to compare emojis with each other (= which emojis are closest in meaning), but also allows us to relate the meaning of emojis with the meaning of other words in the language. Many natural language processing applications depend on the availability of word embeddings. Reliable embeddings for emojis pave the way for many possible uses, starting from being able to rate the semantic similarity of different emoji pairs.

There are, in principle, two ways in which emoji embeddings that are compatible with regular word embeddings (= in the same vector space) can be produced. First, one can treat each emoji in a large corpus as its own token when calculating distributional embeddings for all tokens in a corpus. This essentially provides a distributional representation of each emoji similar to the distributional representations of regular words, and was previously proposed for English by Barbieri et al. (2016). Second, one can use linguistic meaning descriptions of emojis to derive vector representations for them (based on the vectors for the words in the descriptions). For English, Eisner et al. (2016) have used the Unicode names for emojis to derive representations in a word vector space. We compare both types of representations in this section.

The word vector representations provided by spaCy already include word embeddings for 71 of the face emojis in our study. These emoji embeddings were computed together with the regular word embeddings and are in the same vector space; i.e., they were computed following the first method mentioned above. They allow us to compute the similarity between pairs of individual emojis, but also between emojis and other words, using the cosine measure. Presumably, the remainder of the face emojis did not occur frequently enough in the training data to receive their own embedding (only the most frequent 500k tokens are included). We call these distributional vectors “direct emoji embeddings” because they were calculated directly together with the vectors for all other word tokens in the corpus.

In addition, we use a simple heuristic to compute word embeddings based on our elicited emoji descriptions, which we call “description embeddings”. We compute these emoji embeddings by joining all (unlemmatized) meaning descriptions of an emoji together into a document, calculating the overall average of all individual word embeddings of these descriptions, and assigning this average word embedding as the embedding for that emoji. This simple process has many advantages, including that the resulting emoji embedding is relative to the same vector space as embeddings of regular word tokens, and that predominant meanings of an emoji (which are named more often by participants) are weighted strongly. For example, if an (imaginary) emoji receives the descriptions [“happy”, “smiling”, “happy”], we retrieve the embedding vectors for “happy”, “smiling”, and again “happy” and compute the average of them. Thus, the emoji would be located in the vector space in between “happy” and “smiling”, but a bit more closely to “happy”.

We can now compute the cosine “self-similarity” of the direct embedding vector and the description embedding vector for each emoji where both exist. This similarity score expresses how similar the emoji’s use in a corpus is to the use of the words which describe the emoji’s meaning. The self-similarity is relatively low on average, .11 (SD = .06).

**Fig. 9:**
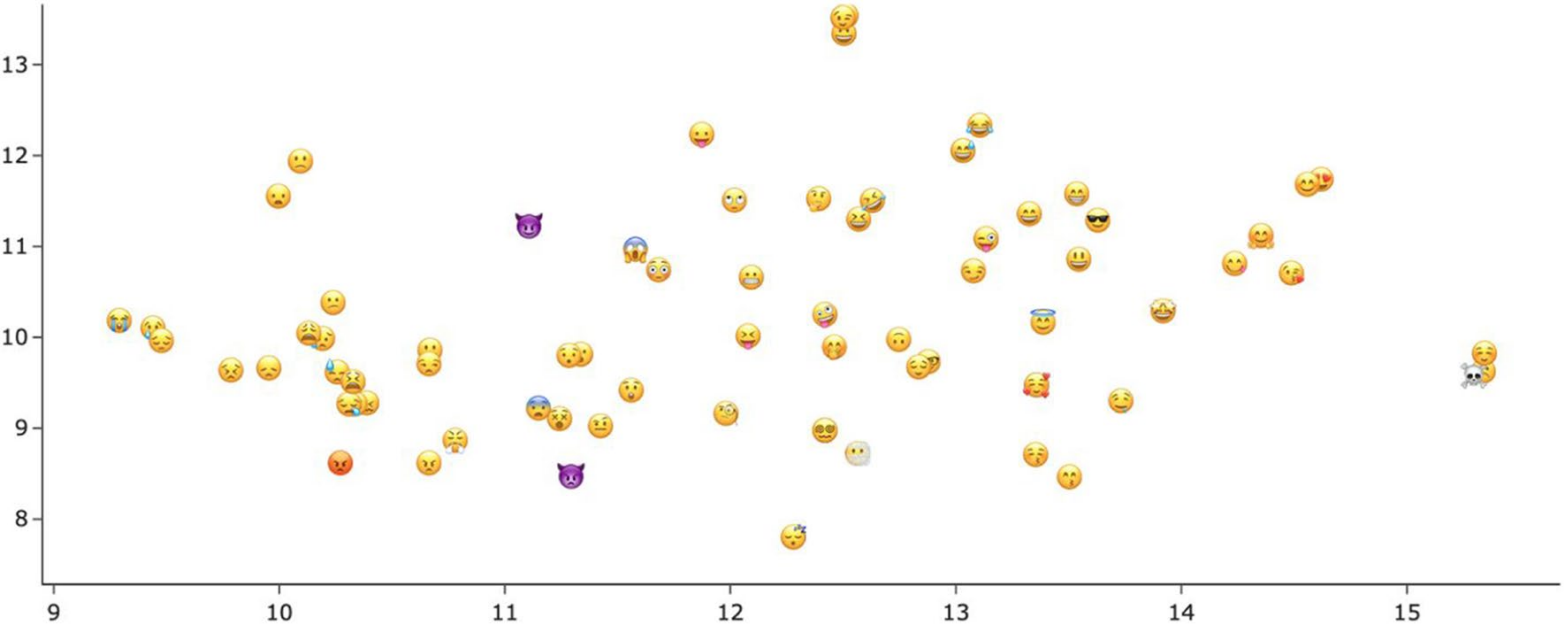
T-SNE plot of direct emoji embeddings

The two kinds of 300 dimensional emoji embeddings themselves cannot be easily directly evaluated, but we can visualize them after dimensionality reduction. We use *t*-distributed stochastic neighbor embedding (t-SNE) to plot the emojis’ vector representations in two main dimensions. T-SNE has the property that relative distances between items are well preserved, so that items that are closer in the high-dimensional space are also visualized near each other in the reduced space (Hinton & Roweis, 2002). Figure 9 depicts the direct emoji embeddings. While many groupings appear reasonable, such as the large group of emojis with frowns and tears on the left side, others seem haphazard. Note for example the overlapping emojis “skull and crossbones”  , “smiling face”  , and “frowning face”  on the very right, which do not seem to share a meaning. Inspecting the nearest neighbors of the face emojis in the word vector space also reveals that the different emojis are only similar to each other, but not closely related to any actual words in the German language. Even the 1000 most similar tokens to an emoji contain no regular word tokens. The entire cloud of emojis is located in its own vector subspace near other non-words: emoticons, laughter expressions (‘haha’, ‘hihihi’), low-frequency terms such as foreign words, and expressions that appear to be hashtags (‘pictureoftheday’). This indicates that the emoji embeddings derived directly from the corpus capture the emojis’ special syntactic properties as tokens that often occur outside regular sentence boundaries. The embeddings do not necessarily capture individual emojis’ “meaning” and their relations amongst each other and with semantically related words. They are therefore less useful to compare emojis with each other semantically.

**Fig. 10:**
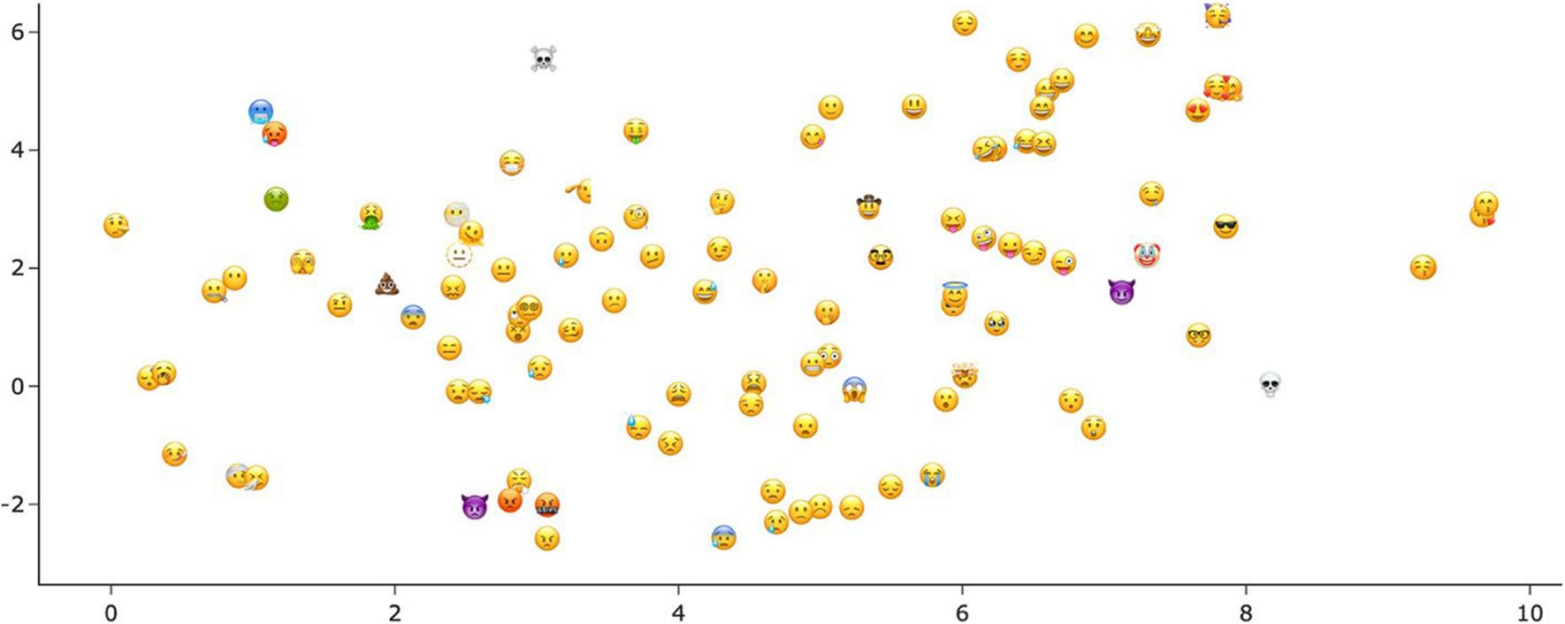
T-SNE plot of emoji description embeddings (average embeddings for our solicited descriptions for each emoji)

In contrast, the description embeddings of all 107 emojis are shown in Fig. 10, reduced to two dimensions. It is clear that emojis with similar meaning components are close to each other in vector space, for example the “hot/cold” emojis at the top left. Even though the two emojis with horns share some part of the meaning (as discussed above), their difference in tone (angry vs. cheeky) causes them to be located in opposite quadrants of the emoji space,  with other “angry” emojis and  near emojis expressing “craziness” or “fun”. Naturally, since the embedding vectors are based on the average of the vectors of the ordinary words used in the emoji descriptions, these emoji description embeddings are also located in the same part of the vector space where those words (‘happy’, ‘evil’, etc., as in Table 4.) appear (see Appendix).

**Fig. 11:**
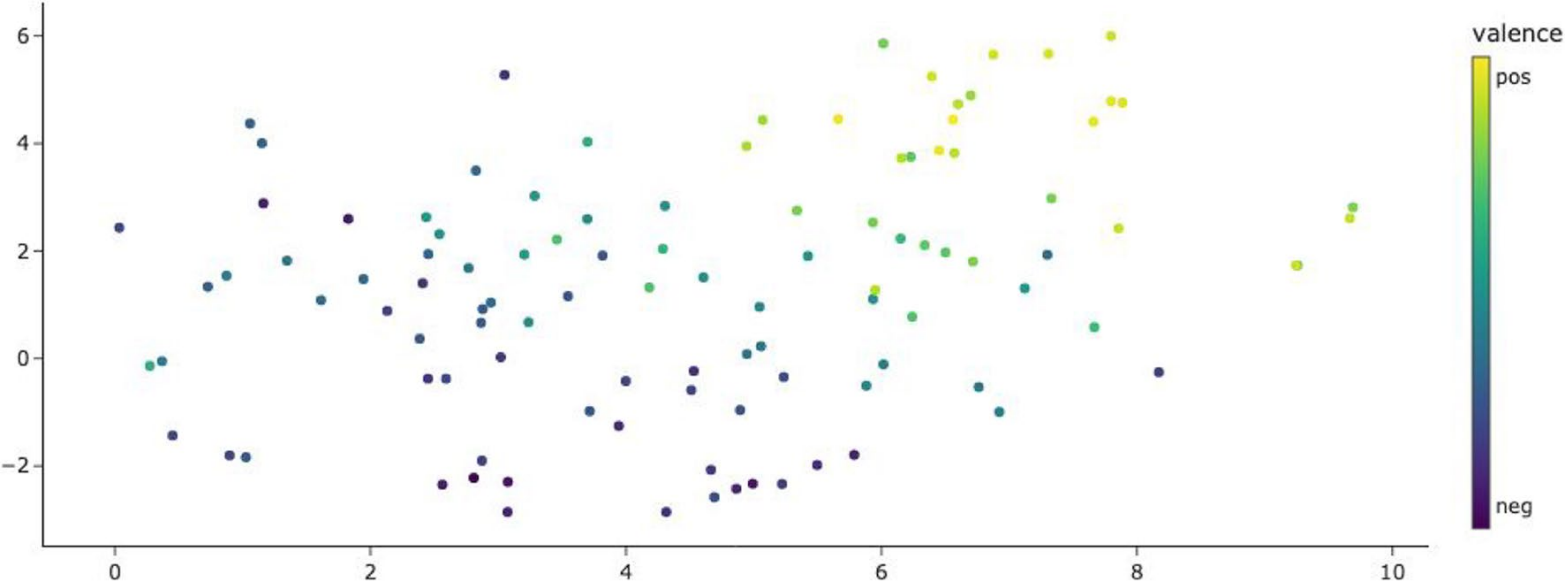
T-SNE plot of emoji description embeddings (identical to Fig. 10), colored by the emoji’s mean valence rating

**Fig. 12:**
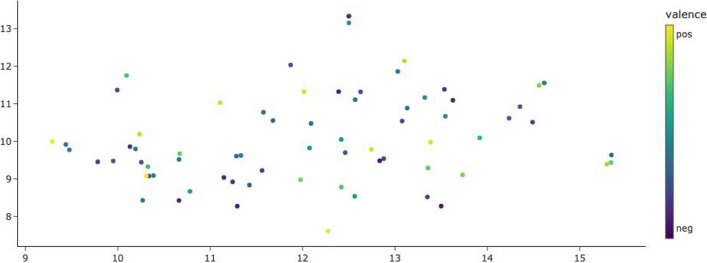
T-SNE plot of direct emoji embeddings (identical to Fig. 9), colored by the emoji’s mean valence rating

Using the description words for the embeddings yields a representation of the emojis’ semantics whose primary axis captures their valence. Figure 11 shows the same t-SNE clustering as Fig. 10, but each emoji is just represented by a dot colored to match its mean valence rating by our study participants. It can be seen that positively rated emojis are located on the (top) right, negatively rated emojis on the left. Emoji valence is thus an important component of their meaning that is also reflected in their meaning descriptions. Figure 12 has the direct emoji embeddings, identical to Fig. 9, colored by mean valence ratings. Here, emoji valence does not correspond to a main component of the emoji vectors.

## Discussion

In the current study, we examined a variety of affective and semantic ratings of 107 face emojis, available at the time, and established norms for arousal, valence, familiarity, clarity, and visual complexity from German-speaking participants. The norms were then correlated with the emojis' frequency of use as determined from private and public German language corpus data. Furthermore, in order to explore the meaning of the emojis, we carried out a description task and collected interpretations of the emojis without contextual cues.

### Validity

The ratings demonstrate strong levels of interrater reliability. In addition, participants used the full rating scale for each measure, and the ratings are distributed near normally, which indicates robust measurement. The validity of our findings is further supported by their positive alignment with the outcomes presented in the two most comprehensive rating studies up to date: Ferré et al. (2023) and Rodrigues et al. (2018). Specifically, strong correlations have been shown in relation to valence across all the datasets we examined. Similarly, the correlation pertaining to arousal between our results and Ferré et al.’s (2023) and Rodrigues et al.’s (2018) work is also high, though slightly less pronounced than the valence correlation. Interestingly, arousal ratings in our study only weakly correlated with the ratings from Kutsuzawa et al. (2022) and were not correlated with the results from Jaeger et al. (2019). We can only speculate that this is due to cultural differences between German and Japanese speakers, alternative arousal definitions, or different rating procedures (Jaeger et al. used a self-assessment manikin to obtain the ratings and do not provide the definition they used in the instruction).

Familiarity, clarity, and visual complexity showed moderate correlations with the ratings from Ferré et al. (2023) and Rodrigues et al. (2018). Several factors could account for the observed pattern of strong correlations in valence, followed by slightly weaker yet still strong correlations in arousal, and moderate correlations in familiarity, clarity, and visual complexity. Valence and arousal appear to generalize well across studies probably because both concepts are easy to grasp by the participants, and closely linked with the meaning of (facial) emojis. Furthermore, particularly arousal could be sensitive to conventionalization and decline over time. The moderate correlations in familiarity, clarity, and visual complexity could stem from the interplay of cultural factors and different study protocols, which make these dimensions more diverse and subject to individual interpretation, thus leading to less consistent associations. A final possibility is that certain measures change over time. One clear example for this is familiarity, which would be expected to increase over time as emojis are used more and in more contexts. In contrast, clarity may decrease if emojis are conventionalized and obtain additional meanings such as the “passive aggressive” sense for the “slightly smiling face”  mentioned by our participants.

To enhance the robustness of our findings, we conducted an additional analysis that delved into the connection between the subjective valence and arousal ratings assigned to the emojis and their meaning descriptions. To achieve this, we leveraged the valence and arousal norms associated with the individual description words given by participants, and averaged these values to generate corresponding estimations for the verbal meaning descriptions of the emojis. Notably, a strong positive correlation emerged between the valence ratings obtained through both direct subjective assessment and via the derived verbal descriptions. This alignment was observed in the consistent pattern where emojis receiving more negative valence ratings were predominantly described using more negative words, and vice versa. Moreover, we identified a moderate to high correlation in the context of arousal. Emojis that garnered higher arousal ratings tended to be accompanied by descriptions containing arousing words.

### Relationships among the measured variables

First, we focus on the interplay between the valence and arousal dimensions of emojis. Replicating a well-documented phenomenon (Kralj Novak et al., 2015; Ferré et al., 2023; Kutsuzawa et al., 2022; Fischer & Herbert, 2021; Jaeger et al., 2019), we observed the emergence of the U-shaped curve. This empirical recurrence underscores that emojis that have high positive or negative valence elicit a more pronounced emotional arousal compared to their emotionally neutral counterparts. Interestingly, our investigation uncovers an asymmetrical facet within this relationship. Emojis with negative valence exhibit on average a higher arousal than positively valenced ones. Li & Wang (2022) report a similar finding for Weibo emojis from Chinese participants. We can speculate that the frequency of emoji use operates as a pivotal mediator. Specifically, the ubiquity of positive emojis in everyday digital communication makes them susceptible to a perceptual desensitization over time, thus attenuating their capacity to elicit intense arousal. This finding mirrors a fundamental relationship between valence and arousal which is not specific for emojis but has also been shown for words or multiword expressions (Citron et al., 2016).

A sharp U-shaped curve can be observed in the relationship between clarity and valence: highly positive or negative face emojis are clearer, while the neutral ones are less clear. This result contrasts with the reported weak positive correlation between valence and clarity in the Portuguese study by Rodrigues et al. (2018). The result can be explained by the frequent use of face emojis to express the emotional evaluation of utterances (Grosz et al., 2023). It may therefore be the case that neutral face emojis are compatible with many different types of situations and thus rated as more ambiguous, vague, or just unclear. Clarity ratings were also mildly negatively correlated with semantic variation from the emoji naming task. This observation confirms the expectation that more ambiguous emojis receive more diverse meaning descriptions.

Subjective familiarity ratings for emojis are moderately correlated with public frequency of use in Twitter, and strongly correlated with an emoji’s frequency in private chats (WhatsApp). This indicates that the subjective rates of exposure correspond relatively well to corpus estimates of frequencies for emojis. Either measure may thus be used in experimental studies to predict participants’ approximate level of exposure to certain emojis. Further, familiarity is significantly positively correlated with clarity; i.e., more familiar emojis are clearer and less confusing. To our knowledge, this is the first study directly comparing familiarity ratings and actual frequency of use from corpus data. In the past, Ferré et al. (2023) compared familiarity and subjective use ratings and reported strong positive correlations between the two. Thus, it appears that familiarity ratings capture both the subjective and objective frequency of use of emojis.

### Impact of the results on experimental investigation of emojis

The results from our study have an impact on stimuli selection and methodology of future studies relating to or using emojis. In particular, the observed correlations between rating measures point towards potential confounds in studies using emojis that depend on their arousal or valence, as these measures are correlated with an emoji’s visual complexity, frequency, and familiarity. Similarly, all studies depending on emoji clarity, such as those asking for sentence interpretations or (graded) grammaticality judgments, or measuring reading time or neurological activity, can be impacted, as clarity is correlated with an emoji’s familiarity and visual complexity.

In particular, in studies comparing only very few emojis which stand in prototypically for certain emotional appraisals (e.g., “loudly crying face”  for ‘sadness’), the selection of those emojis should take care to avoid large differences in frequency, familiarity, and semantic variation between them. Further, even certain well-known emojis have additional senses potentially unexpected by the researcher. For example, while sadness is the dominant meaning of the “loudly crying face”, some participants named other senses such as ‘melodrama’, ‘(overly) cute’ and ‘laughing’. In general, neither relying on the Unicode names of the emojis nor using their Emojipedia definitions to characterize their meaning is in itself sufficient, as the emojis show complex differentiated interpretations in the meaning description part of our study.

In order to better capture the multifaceted emoji meanings, we evaluated two types of distributional representations, so called word embeddings, for the face emojis. The first type are direct emoji embeddings computed from the emojis’ occurrences in natural corpora in the same way as for all other types of word tokens. Our results show that these representations characterize emojis as completely distinct from the regular vocabulary of a language and thus seem to primarily capture emojis’ function as utterance-final items similar to hashtags. The emojis’ meaning and semantic properties (such as valence) are not well represented in these embeddings, and direct emoji embeddings cannot be compared easily with word embeddings for regular word tokens (since the emoji vectors occupy a different part of the semantic space from words). Instead, we proposed a second type of embeddings for the emojis: a simple average of the word embeddings for all tokens used in the elicited meaning descriptions for these emojis. These description embeddings have the advantage that they closely relate emoji representations with word representations, and thus better capture the emojis’ meanings (see Appendix). In consequence, certain meaning components of the emojis (such as their valence) are reflected prominently by the description embeddings.

## Summary and conclusion

In the current study, we collected subjective ratings from German speakers and produced psycholinguistic norms for the set of 107 facial emojis, available at the time of data collection, in five dimensions: visual complexity, familiarity, clarity, emotional valence, and emotional arousal. We also documented each emoji’s frequency of use based on a very large German Twitter corpus, as well as a smaller database of German WhatsApp chats. Finally, we collected verbal meaning descriptions of the emojis and explored them by means of word embeddings.

Our results show that several of the reported measures are correlated, painting a complex picture of emoji interpretation and use. For example, we established that participants’ subjective evaluation of emoji familiarity closely reflects these emojis’ actual objective frequency of use. In return, familiarity with an emoji modulates its valence and clarity. Emoji meanings as established by our description task turn out to be multi-faceted and complex. Many emojis exhibit subtle meaning components not captured by “official” emoji names. In addition, we showed that distributional representations (emoji embeddings) based on the elicited descriptions better capture their semantic relations than direct word2vec based representations.

It must be noted that emojis, like regular words, are not static in their meanings and in their affective associations. Regular use can lead to emojis acquiring additional meanings or losing previous ones. In addition, new emojis are frequently proposed. This affects even the set of face emojis sometimes, as new ones may be added to Unicode. The addition of a new emoji may also lead to shifts in the lexical space that result in the meanings of other emojis changing to accommodate the newcomer. For example, the “dotted line face”  and “melting face”  were both added in 2021. Emotions such as shame or embarrassment may have been expressed using other emojis before, but can be carried by these emojis starting in 2022. This means that the snapshot of current usage of face emojis in the German speaking community reported in this study should be potentially amended by additional investigations in the future or in other linguistic communities.

Our German language emoji norms are the most complete set of measures for face emojis published thus far. The results partially match and expand upon previous rating studies reported for other linguistic backgrounds. We believe that it is important to consider all facets of an emoji’s meaning: both affective values, as well as objective frequency and linguistic descriptions. The relations between the reported measures have an impact on experimental investigation of (utterances with) emojis in at least two ways. First, as previously established for regular words, linguistic processing is affected by specific word qualities like arousal and valence (e.g., Kuperman et al., 2014 for word recognition). Therefore, emoji norms are necessary for future studies using emojis. Second, various properties of emoji stimuli could affect dependent variables in emoji studies. For example, complex emojis are less familiar and clear, which could lead to higher reading times, more errors, or more ambiguous interpretations. Thus, future experiments should carefully control for the variables reported in this paper when selecting stimuli. We hope that the comprehensive emoji norms reported here will facilitate future research on the linguistic and cognitive properties of emojis.

## Figures and Tables

**Fig. 13:**
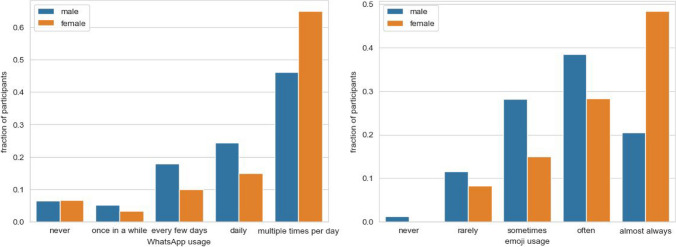
Self-disclosed WhatsApp (*left image*) and emoji usage (*right image*) by gender

**Fig. 14:**
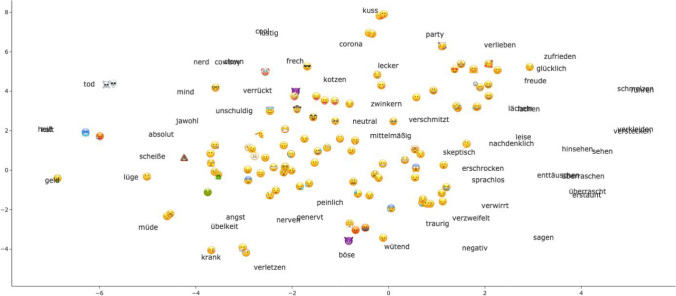
T-SNE plot of emoji description embeddings together with the word embeddings of the most frequently used token describing each emoji

**Fig. 15:**
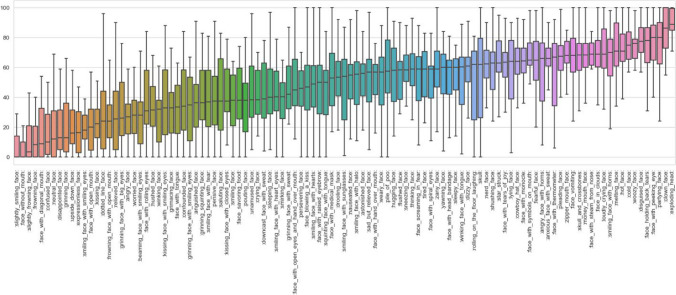
Visual complexity ratings for face emojis

**Fig. 16:**
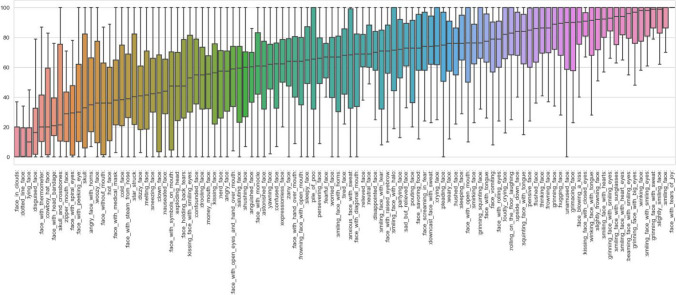
Familiarity ratings for face emojis

**Fig. 17:**
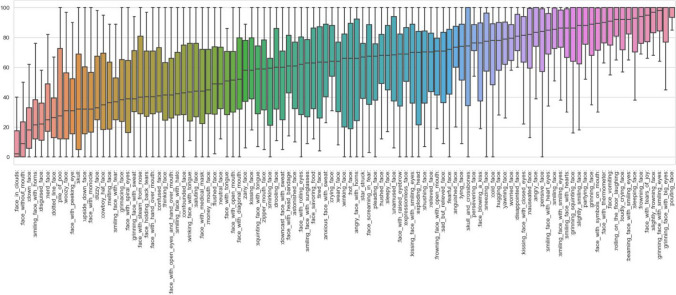
Clarity ratings for face emojis

**Fig. 18:**
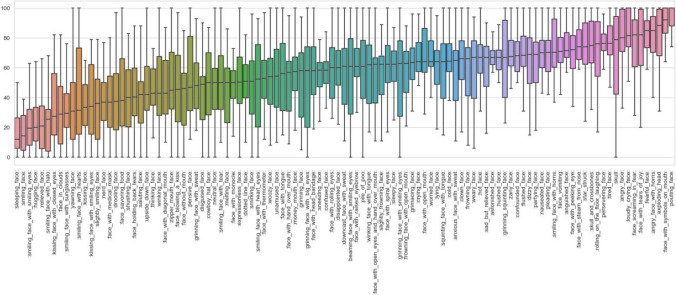
Arousal ratings for face emojis

**Fig. 19:**
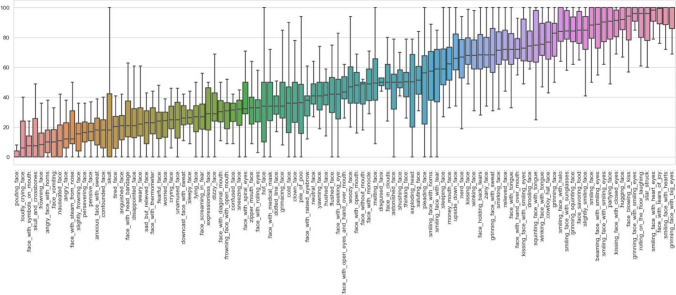
Valence ratings for face emojis

## Data Availability

All stimuli, experimental data and analysis scripts for this study are publicly available via OSF: https://osf.io/vbmpj/. The following columns have been removed from the raw experimental data before upload to protect potentially personal data: “comments”, “experiment id”.

## Footnotes

1 https://unicode.org/emoji/proposals.html#Selection_Factors_Usage

2 Unicode version 15.0, https://www.unicode.org/emoji/charts-15.0/emoji-counts.html

3 https://home.unicode.org/emoji/emoji-frequency/

4 Furthermore, Rodrigues et al. (2018) conducted a direct comparison of ratings between visually distinct Google Android 6.0 and Apple iOS 9.3 emojis, observing significant differences solely in aesthetic appeal, familiarity, clarity, and meaningfulness but not in valence, arousal or visual complexity. Subsequent reanalysis revealed a large effect size discrepancy specifically for only three emojis concerning the clarity dimension. Current emoji designs have converged even more.

5 An anonymous reviewer suggests that arousal may be a variable that is less well-understood by naive raters than valence and that arousal may be influenced by valence ratings if both are carried out at the same time. We see less reason for concern, as (i) other works have also elicited valence before arousal (Fischer & Herbert, 2021), and (ii) our stimuli were presented in blocks by task, so that about 20 other emoji ratings intervene between an emoji’s valence rating and the same emoji’s arousal rating. We thus expect relatively little interference between tasks.

6 https://home.unicode.org/emoji/emoji-frequency

7 All graphs were produced in Python with matplotlib (Hunter, 2007) and seaborn (Waskom, 2021)

8 https://db.mocoda2.de

9 At the suggestion of a reviewer, we performed additional data explorations to check for unattentive participants or those using harmful strategies. We found that all participants responded within a reasonable time frame of on average between 2s and 7s per response, and an inspection of the variability of responses for each rater and numerical rating task did not reveal anomalies. Finally, all participants provided descriptions for almost all emojis, and these descriptions were highly relevant, demonstrating a high motivation to complete the tasks in the manner in which they were intended. We thus did not see the need to exclude further participants beyond the criteria specified above.

10 https://spacy.io/
